# Clinical Spectrum of Oral Secondary Syphilis in HIV-Infected Patients

**DOI:** 10.1155/2013/892427

**Published:** 2012-12-17

**Authors:** Velia Ramírez-Amador, Gabriela Anaya-Saavedra, Brenda Crabtree-Ramírez, Lilly Esquivel-Pedraza, Marcela Saeb-Lima, Juan Sierra-Madero

**Affiliations:** ^1^Departamento de Atención a la Salud, Universidad Autónoma Metropolitana, 04960 Ciudad de México, Mexico; ^2^Clínica de VIH/SIDA, Departamento de Infectología, Instituto Nacional de Ciencias Médicas y Nutrición “Salvador Zubirán”, 14000 Ciudad de México, Mexico; ^3^Departamento de Dermatología, Instituto Nacional de Ciencias Médicas y Nutrición “Salvador Zubirán”, 14000 Ciudad de México, Mexico; ^4^Departamento de Patología, Instituto Nacional de Ciencias Médicas y Nutrición “Salvador Zubirán”, 14000 Ciudad de México, Mexico

## Abstract

*Background*. Oral lesions may constitute the first clinical manifestation in secondary syphilis, but detailed descriptions in HIV-infected individuals are scarce. *Objective*. To describe the clinical characteristics of oral secondary syphilis in HIV-infected patients and its relevance in the early diagnosis of syphilis. *Methods*. Twenty HIV/AIDS adult subjects with oral secondary syphilis lesions presenting at two HIV/AIDS referral centers in Mexico City (2003–2011) are described. An oral examination was performed by specialists in oral pathology and medicine; when possible, a punch biopsy was done, and Warthin-Starry stain and immunohistochemistry were completed. Intraoral herpes virus infection and erythematous candidosis were ruled out by cytological analysis. Diagnosis of oral syphilis was confirmed with positive nontreponemal test (VDRL), and, if possible, fluorescent treponemal antibody test. *Results*. Twenty male patients (median age 31.5, 21–59 years) with oral secondary syphilis lesions were included. Oral lesions were the first clinical sign of syphilis in 16 (80%) cases. Mucous patch was the most common oral manifestation (17, 85.5%), followed by shallow ulcers (2, 10%) and macular lesions (1, 5%). *Conclusions*. Due to the recent rise in HIV-syphilis coinfection, dental and medical practitioners should consider secondary syphilis in the differential diagnosis of oral lesions, particularly in HIV-infected patients.

## 1. Background 

In the USA, since 2001 a resurgence in syphilis incidence, especially among males who have sex with men (MSM), has been observed [1]. By 2004, more than a half of new cases reported of primary and secondary syphilis were estimated to occur in MSM, with a high rate of HIV coinfection [2, 3]. During 2007-2008, the total number of cases of syphilis reported to the CDC increased 13.1% [4]. 

The increasing incidence of syphilis reported in many studies in the last decade, especially among MSM, is clearly a marker for higher risk behavior in this population and raises concerns for a parallel increase in HIV transmission [5–8].

It has been suggested that HIV infection modifies the clinical presentation of syphilis with greater organ involvement, atypical and florid skin rashes, and more rapid progression to neurosyphilis [9–12]; consequently, the diagnosis of syphilis in HIV-infected individuals represents a challenge for care providers. A careful sexual exposure history, recognition of clinical signs and symptoms, and interpretation of diagnostic testing are crucial in this context [13].

Detailed descriptions of oral manifestations of secondary syphilis in HIV-infected individuals are scarce in the scientific literature [14–19], in contrast with several reports in non-HIV-infected individuals [19–34]. Secondary syphilis may mimic other oral lesions such as nonspecific oral ulcerations, oral candidiasis, erythema multiforme, hairy leukoplakia, lichen planus, lupus erythematosus, erythroleukoplakia, and squamous cell carcinoma in both HIV and non-HIV-infected patients [14–34]. Moreover, because of the wide spectrum of oral clinical manifestations occurring in HIV-infected individuals, secondary syphilis in the mouth may be difficult to identify if not suspected by the clinician.

Considering that the incidence of syphilis is increasing in most countries, particularly in HIV-infected individuals [9] and that oral lesions may constitute the first or most florid clinical manifestation of the disease, the aim of the present case series is to describe the clinical spectrum of oral secondary syphilis in HIV-infected patients and its relevance in the early diagnosis of systemic syphilis. 

## 2. Material and Methods

We report a series of 20 consecutive adult subjects with confirmed HIV infection who presented oral secondary syphilis lesions. Patients attended during the period from 2003 to 2011 two HIV/AIDS referral centers in Mexico City: the Instituto Nacional de Ciencias Médicas y Nutrición Salvador Zubirán (INCMNSZ), a third level hospital, and the Clínica Especializada Condesa (CEC), a primary care center dedicated to the health care of HIV/AIDS patients who do not have social security. During the study period, between both institutions, approximately 2,000 active patients were seen.

Specialists in oral pathology and medicine, at the same AIDS clinic visit in both referral centers, consecutively evaluated all HIV-infected patients. During these visits, a structured oral examination was conducted by two of the authors (V. R.-Amador and G. A.-Saavedra). Syphilis-related oral lesions were classified according to previous criteria [35, 36] as follows.

- Macular lesions: flat-to-slightly raised, firm, red lesions with particular predilection of the hard palate.
- Papular lesions: red, raised, firm round nodules with a grey center that may ulcerate, usually located on the buccal mucosa or commissures.
- Mucous patches: slightly raised and covered by a grayish, white pseudomembrane, surrounded by erythema. Lesions appear mainly on the soft palate and pillars, tongue, and vestibular mucosa.
- Shallow ulcers: oval erosions or shallow ulcers of about one cm in diameter, covered by a grey mucoid exudate with an erythematous border.

In order to rule out other oral lesions, lesions suspected as oral syphilis were further evaluated with cytological smear. In macular and papular lesions, it was necessary to consider in the differential diagnosis oral candidosis (OC), particularly the erythematous type. Thus, a cytological smear was taken, and OC was excluded by the absence of *Candida* sp. in periodic acid Schiff (PAS) stained smears and/or the lack of an antifungal treatment response. 

Likewise, in cases of shallow ulcerations located on keratinized oral mucosa, the diagnosis of intraepithelial human herpesvirus infection was ruled out by the absence of virus-infected cells in cytologic smears stained with Papanicolaou, and/or a lack of clinical response to systemic antiviral therapy with acyclovir. 

A punch biopsy from oral lesions was performed in six patients who gave their consent to biopsy. For hematoxylin and eosin (H&E) stain, 5 *μ*m sections were cut from formalin-fixed and paraffin-embedded tissue samples. The silver nitrate-based staining method (Warthin-Starry) was used to identify spirochetes.

Immunohistochemistry was performed using a standard avidin-biotin peroxidase complex technique. Sections were stained with a primary polyclonal antibody against *Treponema pallidum*; antigen-binding sites were visualized through the peroxidase system (Biocare Medical, 4040 Pike Lane, Concord, CA 94520, USA, rabbit polyclonal CAT CP 135B). 

In all patients diagnosis of syphilis was confirmed with a positive nontreponemal test at any titer (Venereal Disease Research Laboratory (VDRL) and if possible, a confirmatory fluorescent treponemal antibody test (FTA-ABS). 

At diagnosis, medical records were reviewed for information about demographic and clinical characteristics which included risk behavior for HIV transmission, clinical stage [37], tobacco and alcohol use, CD4^+^ lymphocyte counts, plasma HIV-RNA level, and current antiretroviral therapy. 

Description of variables was done using the Statistical Program for Social Sciences (SPSS) package, with a 95% confidence level. The prevalence of clinical and laboratory characteristics in patients was reported as percentages. 

## 3. Results

Twenty male adult patients, median age of 31.5 (range 21–59) years with oral secondary syphilis lesions, were identified during the study period, 10 at the AIDS Clinic of the INCMNSZ and 10 at the CEC. The clinical characteristics are shown in Table 1; all patients were MSM, of whom 14 (70%) were receiving HAART, with a median time of use of 32.5 (1–134) months. Of the six patients without HAART, five were naive to antiretrovirals and one had discontinued HAART because of intolerance. The 20 cases were confirmed with positive serologic tests (VDRL in all, FTA-ABS in 15). It is important to mention that CEC is a free governmental HIV/AIDS clinic, so, because of financial issues, FTA-ABS was not performed in five patients; however, in three of them, besides a positive VDRL result, a biopsy led us to confirm the diagnosis. In the other two patients, in addition to a positive VDRL test, the clinical picture was characterized by oral mucous patches which is considered the most typical oral manifestation of secondary syphilis.

In 16 (80%) cases, oral lesions were either the first or most florid clinical sign that led to the diagnosis of secondary syphilis. In the remaining four patients (20%), oral manifestations were part of a systemic clinical picture already diagnosed as secondary syphilis by the treating clinician. 

Eleven of the 20 patients (55%) had mucocutaneous involvement documented in the medical records. Skin manifestations included disseminated maculopapular rash (*n* = 5), hyper pigmented macules/erythematous plaques on palms and soles (*n* = 3), eyebrows/eyelashes alopecia (*n* = 2), and scalp alopecia (1). 

**Figure 1:**
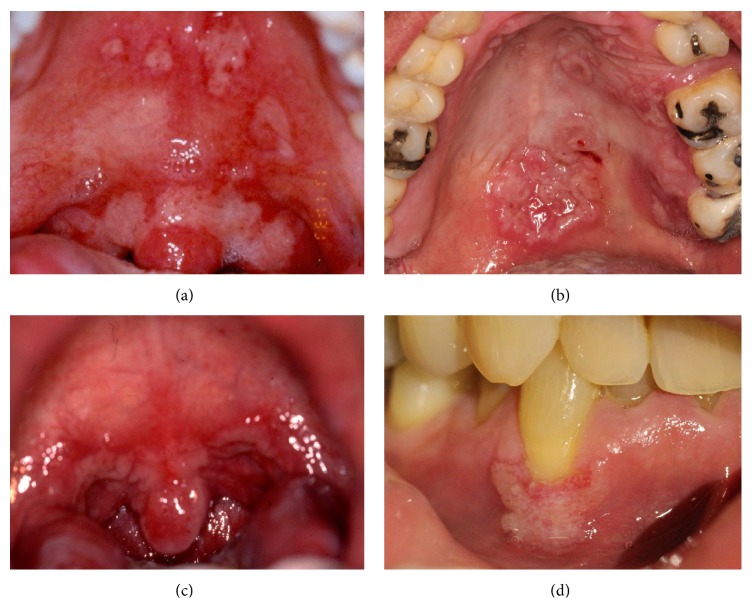
*Oral secondary syphilis.* White slightly raised plaques with a serpentine and white/reddish well-defined outline, located on hard, soft palate, pillars (a–c), and gingiva (d).

The most common clinical manifestation of oral secondary syphilis was mucous patch in 17 cases (85.5%), the soft palate and pillars being the most frequent sites. Mucous patches appeared as white slightly raised plaques on an erythematous base with a serpentine and white/reddish well defined outline (Figure 1). Two patients presented shallow ulcers (10%), and, in one (5%), macular lesions were the most florid oral sign.

Ten (50%) of the 20 patients showed more than one type of syphilis-related oral lesion, concurrent mucous patches and papular lesions being the most frequent combination in four patients, followed by mucous patches and macular lesions in three, and shallow ulcers joint with mucous patches in two and with macular lesions in one (Table 2).

**Figure 2:**
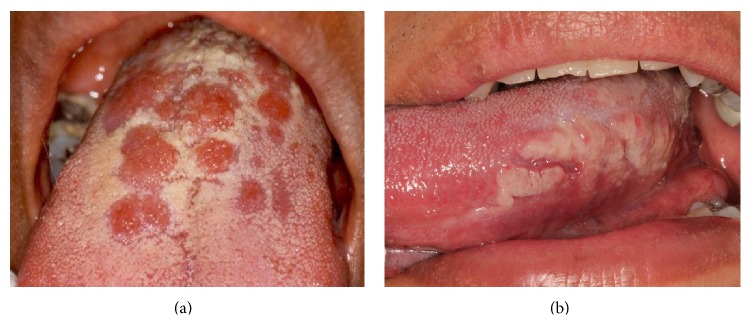
*Oral secondary syphilis.* Papular lesions on the dorsum of the tongue mimicking erythematous candidosis (a), mucous patches, hairy leukoplakia-like, on the lateral side of the tongue (b).

We found four cases (20%) of papular syphilitic lesions in our population, all accompanied with mucous patches and located on dorsal tongue. Macular syphilitic lesions were identified on soft and hard palate (3), dorsum of tongue (1), and labial mucosa (1). In papular and macular cases, the clinical presentation of the oral lesions mimicked erythematous candidosis (Figure 2(a)); the absence of *Candida* sp. hyphae in the PAS smear, and the lack of response to topical or systemic antifungal treatment, allowed us to rule out erythematous candidosis.

**Figure 3:**
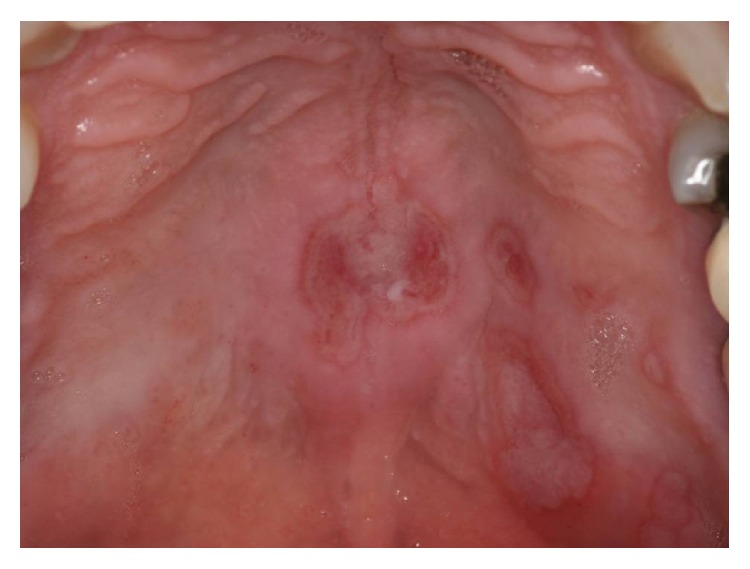
*Oral secondary syphilis.* Shallow ulcers on the hard palate mimicking intraoral herpes virus infection.

One case of mucous patch on the lateral portion of the tongue resembled hairy leukoplakia (Figure 2(b)), and in another one, lesions in both sides of the tongue seemed lichen planus. In both cases a biopsy was taken and based in the histopathological features; the final diagnosis of oral syphilis was confirmed. In the patients who presented shallow ulcers on the palate (cases 1 and 13), the diagnosis of intraoral herpesvirus infection was ruled out as described in the Material and Methods section (Figure 3).

**Figure 4:**
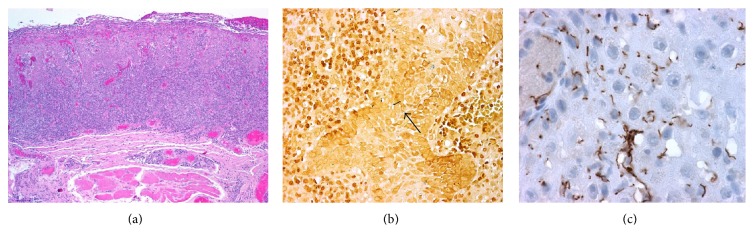
*Secondary syphilis.* H&E stain (4x) shows a psoriasiform and lichenoid lymphoplasmacytic estomatitis with neutrophilic microabscesses in the stratum corneum (a), Warthin Starry stain (10x) shows a spirochete within the epithelium (dark small arrow) (b), and *Treponema pallidum* antibody (20x) shows multiple spirochetes within the epithelium (c).

A biopsy of the oral lesions was taken in six patients who agreed with the procedure; biopsies were processed and stained with H&E and the silver nitrate-based staining method (Warthin-Starry). Microscopically, the lamina propria showed a diffuse (four out of six cases) and a perivascular (three out of six) lymphoplasmacytic inflammatory infiltrate. Occasional eosinophils and neutrophils were also present within the infiltrate and in the epithelium, forming microabscesses. In one case an interface lichenoid pattern was seen. The six oral biopsies examined showed psoriasiform and spongiotic changes in the epithelium; only in one of these cases, a pseudoepitheliomatous hyperplasia was present. Obliteration of the vessels was evident in all cases. Warthin-Starry stain detected spirochetes in the epithelium in two of the six biopsies. Immunohistochemistry for *T. pallidum* revealed numerous spirochetes within keratinocytes, free in the stroma of the lamina propria and within the vessel walls in examined biopsies (Figure 4).

## 4. Discussion

In this paper we report twenty cases of oral secondary syphilis in HIV-infected patients evaluated in two AIDS referral centers in Mexico City, from 2003 to 2011. In most of these cases (80%) the oral lesions were the key clinical finding that led to the diagnosis of secondary syphilis; in the remaining cases, oral findings were additional clinical signs to a well-established clinical picture. 

All cases in this series were males who have sex with men, as it has been stated by several authors in most countries, who have found in recent years an increase in syphilis among MSM [38–42]. In addition, almost half of our patients were young, less than 30 years (45%), in agreement with previous reports [39, 42].

In relation with CD4^+^ cell levels, it was found that only 7 (36.8%) of 19 patients had less than 350 cells/mm^3^ and 6 (30%) had a VL higher than 4 log_10_ copies/mL at the time of syphilis diagnosis. Different studies in HIV-infected patients with syphilis [10, 43–47] have reported a transient decrease in CD4^+^ cell count and an increase in VL. 

It is important to highlight that during the study period no cases of oral primary or tertiary syphilis were observed. As it has been informed in HIV-infected patients [9, 11, 12], a higher rate of secondary syphilis is a common clinical finding; oral lesions are usually seen in secondary disease in patients with HIV [12].

Interestingly, even though the rate of HIV-syphilis coinfection has increased in the recent years [13], reports of oral manifestations of syphilis in HIV-infected patients are scarce [14–19, 48, 49]. In contrast, several reports have described oral lesions in non-HIV individuals [19–34]. 

Regardless of HIV status, the best recognized and characterized oral manifestation of secondary syphilis is the mucous patch, as shown in this case series in which 85.5% of the patients showed the typical slightly raised plaques, mainly located on the palate and soft pillars, which are the common sites described [35, 50]. 

Although the clinical criteria for mucous patches lesions usually involve slightly elevated plaques and superficial ulcers covered by a gray or white pseudomembrane (shallow ulcers) [36], we decided to consider shallow ulcers separately from mucous patches due to two main reasons: the frequent finding of isolated shallow ulcers in our population and the remarkable clinical differences between both types of lesions. Thus, a clinical description for shallow ulcers was added and defined independently from mucous patches as oval erosions or shallow ulcers of about 1 cm in diameter, covered by a grey mucous exudate with an erythematous border. 

In addition to mucous patches [14, 17, 48], shallow ulcers [14, 17], papular [15, 16] and macular lesions [19] have also been described in HIV-infected patients with oral syphilis. As described by Ortega et al. [14, 17] we found four cases of shallow ulcers on palate, tongue, and labial mucosa. Also, as other authors have described [19, 36], palate was the favorite site for macular syphilis lesions that were found in five cases. 

On the other hand, we found four (20%) papular cases, all accompanied with mucous patches and located on dorsal tongue, as it has been described by others [15, 16]. The term papular refers to red, raised, firm round nodules with a grey center that may ulcerate [36]; in this respect, we found that nodular lesions were covered by a depapillated surface, as described by Dalmau et al. [15] and Baniandrés-Rodríguez et al. [16].

The diagnosis of oral secondary syphilis lesions, called the great imitator [24, 51], represents an important clinical challenge. In our report, erythematous candidosis was considered as the differential diagnosis in nine of macular and papular cases, and herpes simplex infection in two patients with shallow ulcers. 

Oral lesions mimicking hairy leukoplakia (HL) or lichen planus (LP) were also observed in one patient each. It is important to explain that in order to confirm the diagnosis of oral syphilis, besides the positive serology test, a biopsy and histopathological analysis were performed to rule out HL and LP. Hairy leukoplakia and leukoplakia-like plaque have been considered in the differential diagnosis of recent case reports of secondary syphilis in HIV seronegative patients [24, 28, 31]. Remarkably, secondary syphilis mimicking pemphigus vulgaris-like oral lesions has been illustrated in a recent case report [22]. 

The definitive diagnosis of syphilis is based on clinical data and complemented with serological and pathological studies. It is important to emphasize that all cases were confirmed with a positive serological test and when possible with histopathological analysis. Demonstration of tissue spirochetes using silver stain (Warthin-Starry) can be difficult, nonspecific, and time consuming, whereas immunohistochemical technique to detect *T. pallidum* in tissue sections has proven to be sensitive and specific and may be very useful in formalin-fixed paraffin wax embedded tissues [52, 53]. 

The present study has certain limitations that need to be taken into account when considering the diagnosis of oral secondary syphilis: first, not all patients gave their consent to the biopsy procedure, and second, there is a lack of confirmatory FTA-ABS tests in five patients of one of the referral centers. However, all five patients had positive VDRL tests, also three had confirmatory histopathological studies and two presented oral mucous patches, which are considered the most typical oral manifestations of secondary syphilis. Also, in doubtful cases a biopsy was done.

Although clinical features (particularly in cases of mucous patches) are highly suggestive of secondary syphilis, VDRL is a nonspecific blood test, so the definitive diagnosis must be confirmed by tissue sample and/or FTA-ABS test. However, it must be considered in agreement with some authors [12, 13, 54] that in secondary syphilis, VDRL is 100% sensitive and that the interpretation of treponemal and nontreponemal serological tests should be equivalent in HIV-infected and non-HIV-infected individuals. Also, in relation with sexual behavior only information about sex preference (MSM or heterosexual) was collected.

Our findings highlight that common oral diseases such as oral candidosis, intraoral human herpes virus infection, hairy leukoplakia, lichen planus, and other conditions considered by others such as lupus erythematosus, erythema multiforme, leukoplakia, erythroleukoplakia, squamous cell carcinoma, and nonspecific erosions should be included in the differential diagnosis of syphilis [14, 15, 17, 19, 24, 25, 27, 31]. 

## 5. Conclusions

Oral manifestations are frequent and protean in HIV-infected patients; lesions caused by syphilis may be easily mistaken for other common entities in these patients, so it is important to emphasize the role of a thorough oral examination in its early diagnosis.

Since syphilis is a treatable disease with a significant potential for serious complications if not treated, a missed diagnosis can have serious consequences for the patient. In routine care, especially in developing countries, accurate diagnostic tools are not always available, so physicians should be conscious that oral lesions could be the only evident clinical manifestation of a common but complex disease like syphilis. 

Clinicians should be aware of the variable manifestations of the disease and the need to suspect it in the presence of oral lesions. Based on the present case series, dental and medical practitioners should consider secondary syphilis in the differential diagnosis of white, ulcerative, popular, and nodular oral lesions, particularly in HIV-infected patients. 

## Figures and Tables

**Table 1:** Clinical characteristics in 20 patients with oral syphilis.

Characteristics	*n* (% )
AIDS^a^	10 (50.0)
On HAART	14 (70.0)
Tobacco consumption (*n* = 19)	6 (31.6)
Alcohol consumption (*n* = 19)	4 (21.1)
Main type of lesion	
Mucous patches	17 (85.5)
Shallow ulcers	2 (10.0)
Macular lesions	1 (5.0)
Oral site of main lesion^b^	
Soft palate-pillars	8 (40.0)
Hard palate	3 (15.0)
Tongue	3 (15.0)
Hard-soft palate-pillars	2 (10.0)
Hard-soft palate-tongue	1 (5.0)
Hard-soft palate-tongue-buccal mucosa	1 (5.0)
Upper labial mucosa	1 (5.0)
Gingiva	1 (5.0)
Minor type of lesion (*n* = 10)	
Papular lesions	4 (40.0)
Macular lesions	4 (40.0)
Shallow ulcers	2 (20.0)
Oral site of minor lesion (*n* = 10)	
Tongue	5 (50.0)
Hard palate	3 (30.0)
Gingiva/labial mucosa	1 (10.0)
Soft palate	1 (10.0)
Median antiretroviral use (range) months (*n* = 14)	32.5 (1–134)
With undetectable viral load (<399 copies/mL) (*n* = 19)	10 (52.6)
Current median CD4^+^ count (range) cells/*μ*L (*n* = 19)	372 (44–734)

^a^ CD4^+^ T-lymphocyte count of <200 cells/*μ*L (or <14 percentage) and/or documentation of an AIDS-defining condition (A3, B3, C1–3) [37]. ^b^Main oral lesion was the most evident and florid oral manifestation.

**Table 2:** Clinical characteristics of 20 patients with oral secondary syphilis.

ID	Clinical stage^a^	Current VL (copies/mL)	Current CD4^+^ (cel/*μ*L)	Oral lesion	Site	VDRL dilution	FTA-ABS
1	B2	6,310	378	Shallow ulcer Macular lesion	Hard palate Gingiva, labial mucosa	1 : 16	++++
2	C3	UND	202	Mucous patches	Hard palate	1 : 8	++
3	C1	NA	NA	Mucous patches	Soft palate-pillars	1 : 32	++++
4	C3	5,470	217	Mucous patches Papular lesion	Soft palate-pillars Dorsal tongue	1 : 256	+++
5	A3	56,500	168	Mucous patches	Soft palate-pillars	1 : 128	+++
6	A2	100,000	239	Mucous patches Papular lesion	Soft palate-pillars Dorsal tongue	1 : 128	+++
7	A1	9,656	681	Shallow ulcers	Upper labial mucosa	1 : 32	NA
8	B3	UND	458	Mucous patches	Soft palate-pillars	1 : 512	+++
9	C3	239,146	44	Macular lesion	Dorsal lingual	1 : 2	+++
10	C3	UND	372	Mucous patches Shallow ulcers	Soft palate-pillars Tongue	1 : 128	+++
11	A1	47,011	603	Mucous patches Macular lesion	Hard palate Hard palate	1 : 2	+
12	A1	13,259	648	Mucous patches Papular lesion	Hard-soft palate-pillars Tongue	1 : 4	+
13	C2	UND	715	Mucous patches Shallow ulcers	Gingiva Hard palate	1 : 64	+++
14	C2	UND	360	Mucous patches	Hard-soft palate-tongue	1 : 32	NA
15	C3	UND	187	Mucous patches Papular lesion	Soft palate-pillars Dorsal tongue	1 : 128	+++
16	C3	UND	348	Mucous patches Macular lesion	Soft palate-pillars, tongue Hard palate	1 : 32	NA
17	B3	UND	370	Mucous patches	Lateral sides of tongue	1 : 32	NA
18	C3	UND	392	Mucous patches	Soft palate-pillars	1 : 32	NA
19	A1	UND	734	Mucous patches	Hard-soft palate-pillars	1 : 64	+++
20	A1	205,000	532	Mucous patches Macular lesion	Lateral tongue Soft palate	1 : 64	++

ID: identification, ^a^according to the CDC revised surveillance case definitions [37], VL: viral load, VDRL: venereal disease research laboratory, FTA-ABS: fluorescent treponemal antibody absorbed, UND: undetectable, NA: not available.

## References

[1] Buchacz K., Klausner J. D., Kerndt P. R. (2008). HIV incidence among men diagnosed with early syphilis in Atlanta, San Francisco, and Los Angeles, 2004 to 2005. *Journal of Acquired Immune Deficiency Syndromes*.

[2] Centers for Disease Control and Prevention (CDC) (2005). *Sexually Transmitted Disease Surveillance 2004 Supplement: Syphilis Surveillance Report*.

[3] Centers for Disease Control and Prevention (CDC) (2006). Primary and secondary syphilis—United States, 2003-2004. *Morbidity and Mortality Weekly Report*.

[4] Centers for Disease Control and Prevention (CDC) (2009). *Sexually Transmitted Disease Surveillance, 2008*.

[5] Ganesan A., Fieberg A., Agan B. K. (2012). Results of a 25-year longitudinal analysis of the serologic incidence of syphilis in a cohort of HIV-infected patients with unrestricted access to care. *Sexually Transmitted Diseases*.

[6] Centers for Disease Control and Prevention (2011). *Sexually Transmitted Disease Surveillance 2010*.

[7] Bremer V., Marcus U., Hamouda O. (2012). Syphilis on the rise again in Germany—results from surveillance data for 2011. *Eurosurveillance*.

[8] Li D., Jia Y., Ruan Y. (2010). Correlates of incident infections for HIV, syphilis, and hepatitis B virus in A cohort of men who have sex with men in Beijing. *AIDS Patient Care and STDs*.

[9] Karp G., Schlaeffer F., Jotkowitz A., Riesenberg K. (2009). Syphilis and HIV co-infection. *European Journal of Internal Medicine*.

[10] Zetola N. M., Klausner J. D. (2007). Syphilis and HIV infection: an update. *Clinical Infectious Diseases*.

[11] Pialoux G., Vimon S., Moulignier A., Buteux M., Abraham B., Bonnard P. (2008). Effect of HIV infection on the course of syphilis. *AIDS Reviews*.

[12] Lynn W. A., Lightman S. (2004). Syphilis and HIV: a dangerous combination. *Lancet Infectious Diseases*.

[13] Zetola N. M., Engelman J., Jensen T. P., Klausner J. D. (2007). Syphilis in the United States: an update for clinicians with an emphasis on HIV coinfection. *Mayo Clinic Proceedings*.

[14] Ortega K. L., Rezende N. P. M., Magalhães M. H. C. G. (2009). Diagnosing secondary syphilis in a patient with HIV. *British Journal of Oral and Maxillofacial Surgery*.

[15] Dalmau J., Alegre M., Sambeat M. A., Roé E., Peramiquel L., Alomar A. (2006). Syphilitic nodules on the tongue. *Journal of the American Academy of Dermatology*.

[16] Baniandrés Rodríguez O., Nieto Perea O., Moya Alonso L., Carrillo Gijón R., Harto Castaño A. (2004). Nodular secondary syphilis in a HIV patient mimicking cutaneous lymphoma. *Anales de Medicina Interna*.

[17] Ortega K. L., Rezende N. P., Watanuki F., Soares De Araujo N., Magalhaes M. H. C. G. (2004). Secondary syphilis in an HIV positive patient. *Medicina Oral*.

[18] Romero-Jiménez M. J., Suárez Lozano I., Fajardo Picó J. M., Barón Franco B. (2003). Malignant syphilis in patient with human immunodeficiency virus (HIV): case report and literature review. *Anales de Medicina Interna*.

[19] Lu S. Y., Eng H. L. (2002). Secondary syphilis-related oral ulcers: report of four cases. *Chang Gung Medical Journal*.

[20] Czerninski R., Pikovski A., Meir K., Casap N., Moses A. E., Maly A. (2011). Oral syphilis lesions—a diagnostic approach and histologic characteristics of secondary stage. *Quintessence International*.

[21] Ikenberg K., Springer E., Bräuninger W. (2010). Oropharyngeal lesions and cervical lymphadenopathy: syphilis is a differential diagnosis that is still relevant. *Journal of Clinical Pathology*.

[22] Mignogna M. D., Fortuna G., Leuci S., Mignogna C., Delfino M. (2009). Secondary syphilis mimicking pemphigus vulgaris. *Journal of the European Academy of Dermatology and Venereology*.

[23] Murrell G. L. (2009). Secondary syphilis oral ulcer. *Otolaryngology-Head and Neck Surgery*.

[24] Compilato D., Amato S., Campisi G. (2009). Resurgence of syphilis: a diagnosis based on unusual oral mucosa lesions. *Oral Surgery, Oral Medicine, Oral Pathology, Oral Radiology and Endodontology*.

[25] Carlesimo M., Palese E., Mari E. (2008). Isolated oral erosions: an unusual manifestation of secondary syphilis. *Dermatology Online Journal*.

[26] Hayes M., White D., Richards A. (2008). Secondary syphilis presenting as atypical oral ulceration—a case report. *Dental Update*.

[27] Herrero-gonzález J. E., Amer M. E. P., Farrés M. F., Abelló A. T., Barranco C., Pujol R. M. (2008). Syphilitic mucous patches: the resurgence of an old classic. *International Journal of Dermatology*.

[28] Stepanova A., Marsch W. C. (2006). Plaques opalines. A rare form of secondary syphilis of the oral mucous membrane. *Hautarzt*.

[29] Hua H., Yan Z. M., Shi R. T., Gao Y., Xu Y. Y. (2005). Clinical and pathological analysis of oral manifestations of 40 patients with secondary syphilis. *Zhonghua Kou Qiang Yi Xue Za Zhi*.

[30] Paz A., Potasman I. (2004). Oral lesions as the sole presenting symptom of secondary syphilis. *Travel Medicine and Infectious Disease*.

[31] Aquilina C., Viraben R., Denis P. (2003). Secondary syphilis simulating oral hairy leukoplakia. *Journal of the American Academy of Dermatology*.

[32] Christen A., Killer H. E., Stamm B., Itin P. (2003). Secundary syphilis with ocular manifestation and oral ulceration. *Schweizerische Rundschau fur Medizin-Praxis*.

[33] Dave S., Gopinath D. V., Thappa D. M. (2003). Nodular secondary syphilis. *Dermatology Online Journal*.

[34] Ulmer A., Fierlbeck G. (2002). Images in clinical medicine. Oral manifestations of secondary syphilis. *The New England Journal of Medicine*.

[35] Ficarra G., Carlos R. (2009). Syphilis: the renaissance of an old disease with oral implications. *Head and Neck Pathology*.

[36] Leão J. C., Gueiros L. A., Porter S. R. (2006). Oral manifestations of syphilis. *Clinics*.

[37] Schneider E., Whitmore S., Glynn K. M., Dominguez K., Mitsch A., McKenna M. T. (2008). Revised surveillance case definitions for HIV infection among adults, adolescents, and children aged <18 months and for HIV infection and AIDS among children aged 18 months to <13 years—United States, 2008. *Morbidity and Mortality Weekly Report*.

[38] Adolf R., Bercht F., Aronis M. L., Lunardi L. W., Schechter M., Sprinz E. (2012). Prevalence and risk factors associated with syphilis in a cohort of HIV positive individuals in Brazil. *AIDS Care*.

[39] Thurnheer M. C., Weber R., Toutous-Trellu L. (2010). Occurrence, risk factors, diagnosis and treatment of syphilis in the prospective observational Swiss HIV Cohort Study. *AIDS*.

[40] Chow E. P., Wilson D. P., Zhang L. (2011). HIV and syphilis co-infection increasing among men who have sex with men in China: a systematic review and meta-analysis. *PLoS One*.

[41] Muldoon E., Mulcahy F. (2011). Syphilis resurgence in Dublin, Ireland. *International Journal of STD & AIDS*.

[42] Pathela P., Braunstein S. L., Schillinger J. A., Shepard C., Sweeney M., Blank S. (2011). Men who have sex with men have a 140-fold higher risk for newly diagnosed HIV and syphilis compared with heterosexual men in New York City. *JAIDS Journal of Acquired Immune Deficiency Syndromes*.

[43] Jarzebowski W., Caumes E., Dupin N. (2012). Effect of early syphilis infection on plasma viral load and CD4 cell count in human immunodeficiency virus-infected men: results from the FHDH-ANRS CO4 cohort. *Archives of Internal Medicine*.

[44] Weintrob A. C., Gu W., Qin J. (2010). Syphilis co-infection does not affect HIV disease progression. *International Journal of STD and AIDS*.

[45] Palacios R., Jiménez-Oñate F., Aguilar M. (2007). Impact of syphilis infection on HIV viral load and CD4 cell counts in HIV-infected patients. *Journal of Acquired Immune Deficiency Syndromes*.

[46] Kofoed K., Gerstoft J., Mathiesen L. R., Benfield T. (2006). Syphilis and human immunodeficiency virus (HIV)-1 coinfection: influence on CD4 T-cell count, HIV-1 viral load, and treatment response. *Sexually Transmitted Diseases*.

[47] Buchacz K., Patel P., Taylor M. (2004). Syphilis increases HIV viral load and decreases CD4 cell counts in HIV-infected patients with new syphilis infections. *AIDS*.

[48] Ramirez-Amador V., Sierra Madero J. G., Pedraza L. E. (1996). Oral secondary syphilis in a patient with human immunodeficiency virus infection. *Oral Surgery, Oral Medicine, Oral Pathology, Oral Radiology, and Endodontics*.

[49] Ficarra G., Zaragoza A. M., Stendardi L., Parri F., Cockerell C. J. (1993). Early oral presentation of lues maligna in a patient with HIV infection: a case report. *Oral Surgery Oral Medicine and Oral Pathology*.

[50] Bruce A. J., Rogers R. S. (2004). Oral manifestations of sexually transmitted diseases. *Clinics in Dermatology*.

[51] Domantay-Apostol G. P., Handog E. B., Gabriel M. T. G. (2008). Syphilis: the international challenge of the great imitator. *Dermatologic Clinics*.

[52] Buffet M., Grange P. A., Gerhardt P. (2007). Diagnosing Treponema pallidum in secondary syphilis by PCR and immunohistochemistry. *Journal of Investigative Dermatology*.

[53] Barrett A. W., Dorrego M. V., Hodgson T. A. (2004). The histopathology of syphilis of the oral mucosa. *Journal of Oral Pathology and Medicine*.

[54] Daskalakis D. (2008). Syphilis: continuing public health and diagnostic challenges. *Current HIV/AIDS Reports*.

